# Are the Sexual and Reproductive Health Care Needs of Premenopausal Female Service Users Accessing Early Intervention in Psychosis Services Being Adequately Addressed?

**DOI:** 10.1192/bjo.2025.10430

**Published:** 2025-06-20

**Authors:** Sophie Ratcliffe

**Affiliations:** Avon and Wiltshire Partnership NHS Trust, Bristol, United Kingdom

## Abstract

**Aims:** Sexual and reproductive health (SRH) is often overlooked in the care of individuals with severe mental illness, despite national guidance from NHS England advocating its inclusion in routine mental health care. Women with psychosis are at increased risk of relapse during key life stages such as pregnancy, childbirth, and menopause. They often face compounded vulnerabilities, including intimate partner violence, substance misuse, and barriers to accessing reliable contraception. This audit aimed to assess whether the SRH needs of premenopausal female service users are considered and addressed within South Gloucestershire’s Early Intervention in Psychosis (EI) service.

**Methods:** 50 females were receiving care from South Gloucestershire EI service in December 2024. Their electronic records were reviewed retrospectively. 13 individuals were excluded for the following reasons: 11 post-menopausal, 1 primary ovarian insufficiency, 1 transgender. The remaining 37 pre-menopausal females were included in the audit. Their mean age was 33, range 17–48 years. Their Personal Wellbeing Plans, clinical documents, physical health assessments, primary care contraception prescriptions, and clinical progress notes were searched for evidence of SRH discussions relating to contraception, sexually transmitted infection (STI) screening, and signposting to SRH community services.

**Results:** Documentation of discussions about contraception and STI screening were minimal, found in 0% of physical health assessments, 3% of Personal Wellbeing Plans, 11% of clinical letters, and 5% of electronic progress notes (all post high-risk events). Uptake of long-acting reversible contraceptives was low with only 8% accessing IUS, 0% IUD and 5% implants.

The women displayed notable vulnerabilities: 51% had experienced intimate partner violence, 62% had a history of sexual assault (27% in past year), 46% reported substance misuse, and there were child safeguarding concerns in 38%, with 16% having children no longer in their care. Two unplanned pregnancies had occurred in the past year. From a mental health perspective, 11% had previous perinatal or postnatal mental illness, and 5% had been admitted to specialist mother-and-baby units.

**Conclusion:** There is a critical gap in addressing the SRH needs of females accessing the EI psychosis service. Despite the high risks, SRH discussions and referrals to community services were infrequent. Care could be enhanced by staff training, introduction of routine screening for SRH needs on intake to the service, and at reviews, and inclusion into health checks, supported by clearer referral pathways to access community SRH services. These improvements are crucial to meeting national and local guidance and improving SRH and mental health outcomes for this vulnerable group.

